# Pericoronary adipose tissue attenuation on coronary computed tomography angiography associates with male sex and Indigenous Australian status

**DOI:** 10.1038/s41598-023-41341-9

**Published:** 2023-09-19

**Authors:** Jeremy Yuvaraj, Egynne Lim, Tony Vo, David Huynh, Cheniqua Rocco, Nitesh Nerlekar, Kevin Cheng, Andrew Lin, Damini Dey, Stephen J. Nicholls, Nadarajah Kangaharan, Dennis T.L. Wong

**Affiliations:** 1 Monash Cardiovascular Research Centre, Victorian Heart Institute, MonashHeart and Monash University, Monash Health, 246 Clayton Road, Clayton, VIC 3168 Australia; 2 School of Clinical Sciences, Monash University, Clayton, VIC Australia; 3 Division of Medicine, Royal Darwin Hospital, Tiwi, NT Australia; 4 Department of Cardiology, Gold Coast University Hospital, Southport, QLD Australia; 5 Cedars-Sinai Medical Center, Biomedical Imaging Research Institute, Los Angeles, CA USA

**Keywords:** Inflammation, Translational research, Cardiovascular diseases

## Abstract

To evaluate if Indigenous Australians have higher coronary inflammation demonstrated non-invasively using pericoronary adipose tissue attenuation on coronary computed tomography angiography (CCTA). We retrospectively obtained a cohort 54 Indigenous patients age- and sex-matched to 54 non-Indigenous controls (age: 46.5 ± 13.1 years; male: n = 66) undergoing CCTA at the Royal Darwin Hospital and Monash Medical Centre. Patient groups were defined to investigate the interaction of ethnicity and sex: Indigenous + male, Indigenous + female, control + male, control + female. Semi-automated software was used to assess pericoronary adipose tissue attenuation (PCAT-a) and volume (PCAT-v). Males had significantly higher PCAT-a (– 86.7 ± 7.8 HU vs. − 91.3 ± 7.1 HU, p = 0.003) than females. Indigenous patients had significantly higher PCAT-v (1.5 ± 0.5cm^3^ vs. 1.3 ± 0.4cm^3^, p = 0.032), but only numerically higher PCAT-a (p = 0.133) than controls. There was a significant difference in PCAT-a and PCAT-v across groups defined by Indigenous status and sex (p = 0.010 and p = 0.030, respectively). Among patients with matching CCTA contrast density, multivariable linear regression analysis showed an independent association between Indigenous status and PCAT-a. Indigenous men have increased PCAT-a in an age- and sex-matched cohort. Male sex is strongly associated with increased PCAT-a. Coronary inflammation may contribute to adverse cardiovascular outcomes in Indigenous Australians, but larger studies are required to validate these findings.

## Introduction

Coronary artery disease is a prominent cause of mortality worldwide and accounts for the leading cause of death in Australian men. Indigenous Australians face disproportionately poorer cardiovascular outcomes, being at three times greater risk of major coronary events, and 1.5 times greater risk of mortality from these events^1^. This may be owed in part to a combination of higher incidence of traditional risk factors and chronic comorbidities, in combination with socioeconomic barriers impeding access to primary prevention and routine cardiovascular care, but it is unlikely that these elements alone account for the vast disparity in events and mortality between Indigenous and non-Indigenous Australians^1,2^. In addition to these findings, Indigenous Australians also harbour increased epicardial adipose tissue (EAT) volume^3^, indicating excess cardiac adiposity is a component in the adverse cardiometabolic profile faced by Indigenous Australians.

Pericoronary adipose tissue (PCAT) lies directly adjacent to the coronary adventitia and is an established surrogate marker of coronary inflammation. Increased attenuation of this depot on routine coronary computed tomography angiography (CCTA) indicates phenotypic changes to adipocyte morphology resultant of inflammatory mediators originating from the adjacent vessel bed, and is associated with vulnerable plaque development and coronary events^4–6^. Elevated C-reactive protein (CRP) has been previously reported in Indigenous Australians and associates with the presence of renal dysfunction and cardiovascular disease specifically within this group^7–9^. PCAT attenuation, however, has not been explored amongst Indigenous Australians, and thus the degree to which local coronary inflammation may be implicated in the increased cardiovascular risk faced by this ethnic group has yet to be ascertained. We therefore aimed to evaluate coronary inflammation in a matched cohort of Indigenous Australians.

## Results

### Baseline characteristics

**Figure 1:**
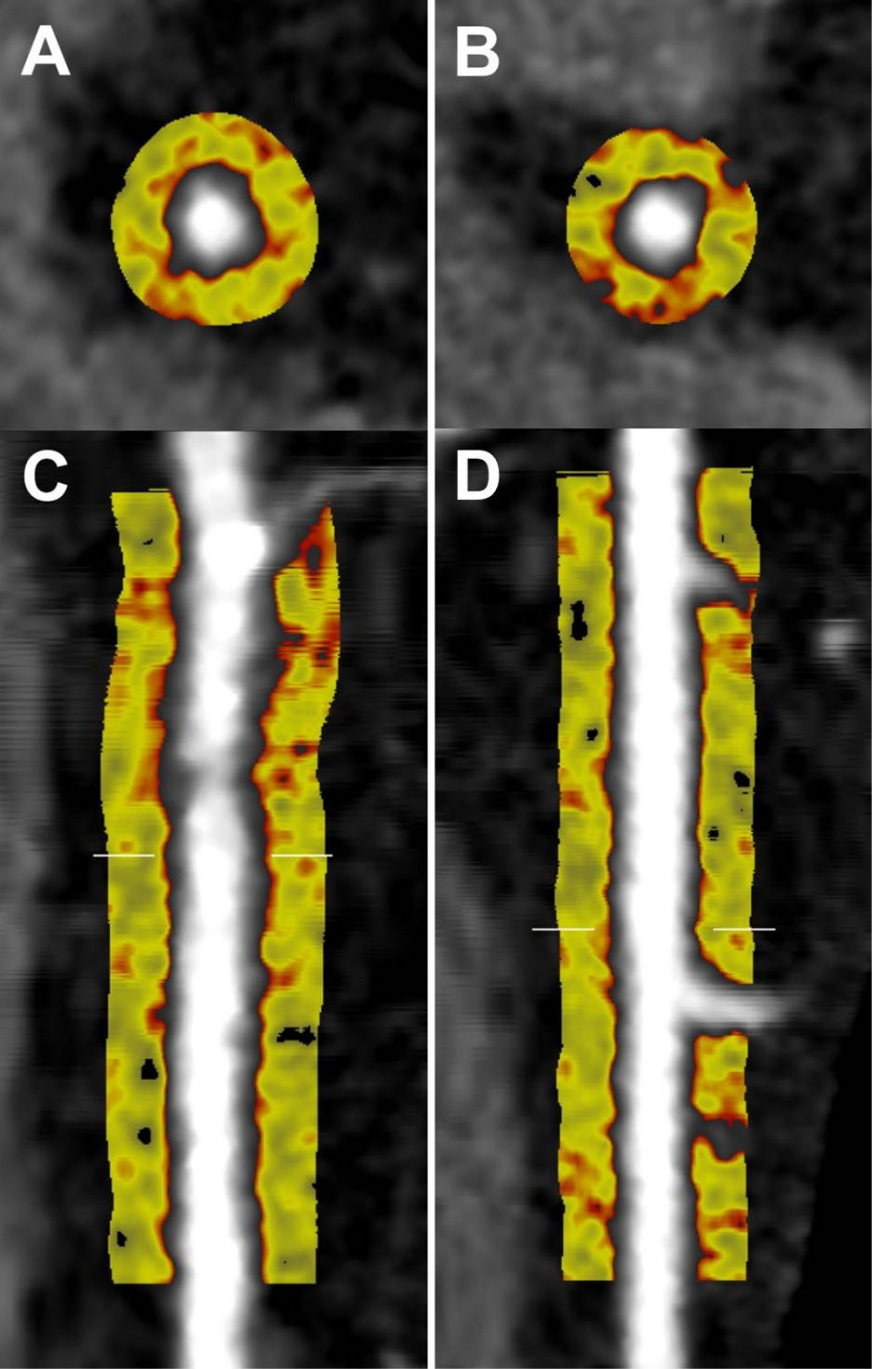
Case comparison of PCAT segmentation in the proximal RCA in an Indigenous Australian patient (left panel) and non-Indigenous patient (right panel). PCAT is represented on cross-sectional (**A**,**B**) and longitudinal views (**C**,**D**). Colour map highlights PCAT attenuation in HU ranging from − 190 HU (yellow) to − 30 HU (red). *PCAT* pericoronary adipose tissue, *RCA* right coronary artery, *HU* hounsfield units.

Baseline patient characteristics are summarised in Table 1. A total of 108 patients were studied (age: 46.5 ± 13.1 years; 61.1% male). Smoking was more prevalent among Indigenous patients than controls (49.0% vs. 29.6%, p = 0.042). There were no other significant differences in cardiovascular risk factors or statin use between Indigenous patients and controls (all p > 0.05). Indigenous patients had significantly higher prevalence of obstructive CAD (40.0% vs. 13.7%, p = 0.003); there were no other differences in CAD prevalence, SIS, SSS, and CT-LeSc (all p > 0.05, Table 1). Mean PCAT-a within the overall cohort was − 88.5 ± 7.8 HU, and mean PCAT-v was 1.4 ± 0.4 cm^3^. PCAT-a in an Indigenous patient and a control patient is represented visually in Fig. 1.

**Table 1:** Baseline characteristics.

	Indigenous	Control	*p*-value
Cardiovascular risk factors, *n* (%)			
Hypertension	20 (39.2)	22 (40.7)	0.873
Hypercholesterolaemia	17 (33.3)	22 (40.7)	0.432
Smoker	25 (49.0)	16 (32.0)	0.042
Type II diabetes	12 (23.5)	6 (11.1)	0.092
Obesity	12 (26.1)	14 (28.0)	0.833
CAD, *n* (%)	30 (60.0)	25 (49.0)	0.268
Obstructive CAD	20 (40.0)	7 (31.7)	0.003
Plaque burden, median (IQR)			
SIS	1.5 (0.0, 6.9)	0.0 (0.0, 3.0)	0.145
SSS	2.5 (0.0, 11.3)	0.0 (0.0, 5.0)	0.082
CT-Leaman score, *n* (%)	2.7 (0.0, 9.4)	0.0 (0.0, 6.5)	0.120
No plaque	20 (40.0)	26 (51.0)	0.121
Low plaque burden	16 (32.0)	19 (37.3)	
High plaque burden	14 (28.0)	6 (11.8)	
Statin, *n* (%)	15 (31.3)	18 (33.3)	0.822

All proportions are of modified totals excluding missing data.

### Relationship between PCAT and plaque burden

PCAT-a was not correlated with SIS (r = 0.136, p = 0.176), SSS (r = 0.158, p = 0.116) or CT-LeSc (r = 0.164, p = 0.101). PCAT-v positively correlated with SIS (r = 0.294, p = 0.003), SSS (r = 0.288, p = 0.003) and CT-LeSc (r = 0.239, p = 0.016). PCAT-a was not significantly different across categories of plaque burden (no plaque, − 89.2 ± 8.5 HU; low plaque burden, − 90.3 ± 6.1 HU; high plaque burden, − 85.4 ± 8.6 HU; p = 0.072). PCAT-v was significantly different between categories of plaque burden (no plaque, 1.2 ± 0.4cm^3^; low plaque burden, 1.5 ± 0.5cm^3^; high plaque burden, 1.5 ± 0.4cm^3^; p = 0.003), with significant pairwise differences between no plaque and low plaque burden (p = 0.011) and no plaque and high plaque burden (p = 0.014). PCAT-a was significantly higher in patients with obstructive CAD (− 85.5 ± 7.7 vs. − 89.7 ± 8.0 HU, p = 0.030). PCAT-v was not significantly different in patients with obstructive CAD (1.5 ± 0.4cm^3^ vs. 1.3 ± 0.4cm^3^, p = 0.063).

### Relationship between PCAT and patient characteristics

**Figure 2:**
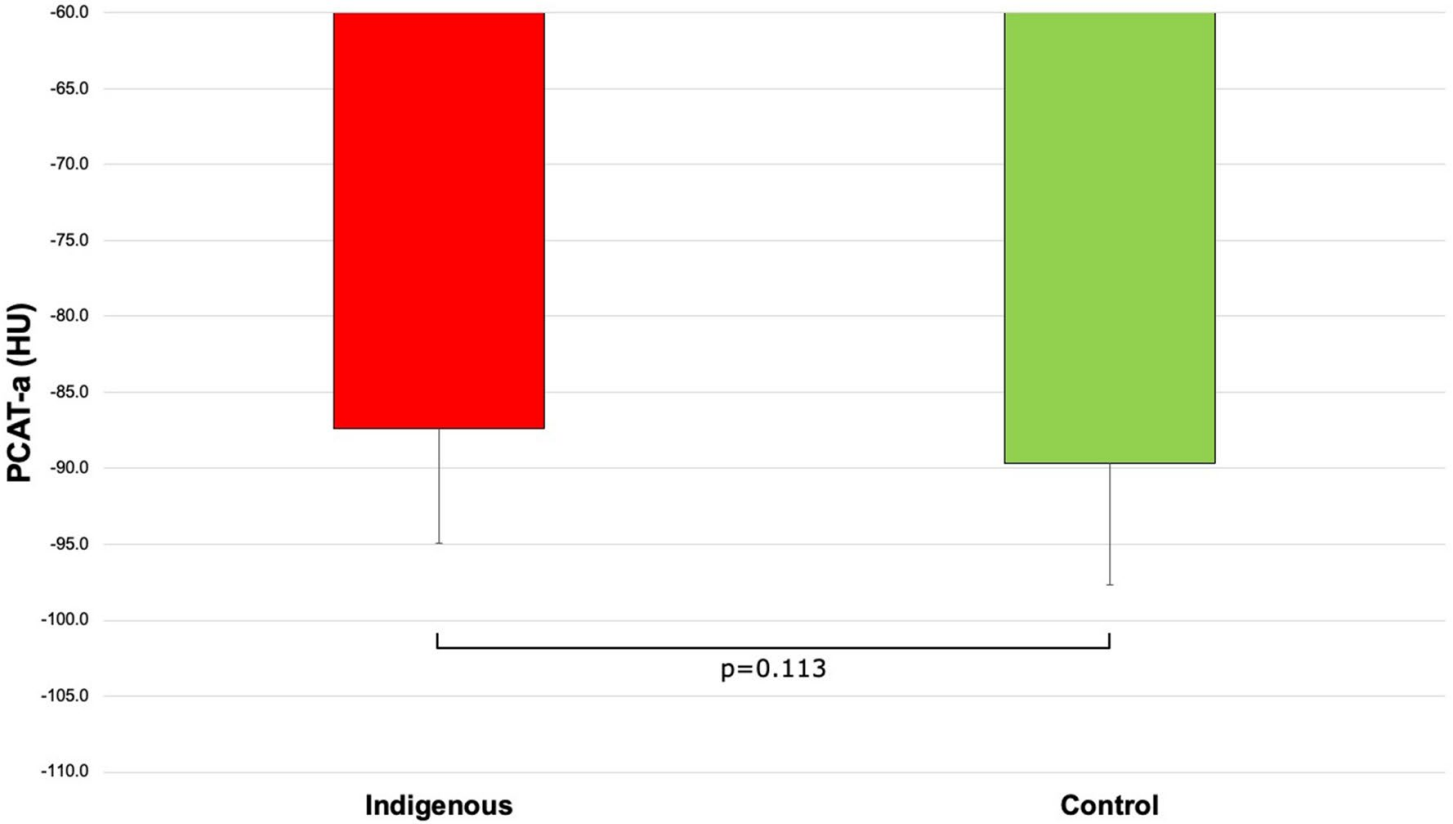
Comparison of PCAT-a in indigenous Australians versus controls (− 87.4 ± 7.6 HU vs. − 89.6 ± 8.0 HU, p = 0.133). *PCAT-a* PCAT-attenuation.

**Figure 3:**
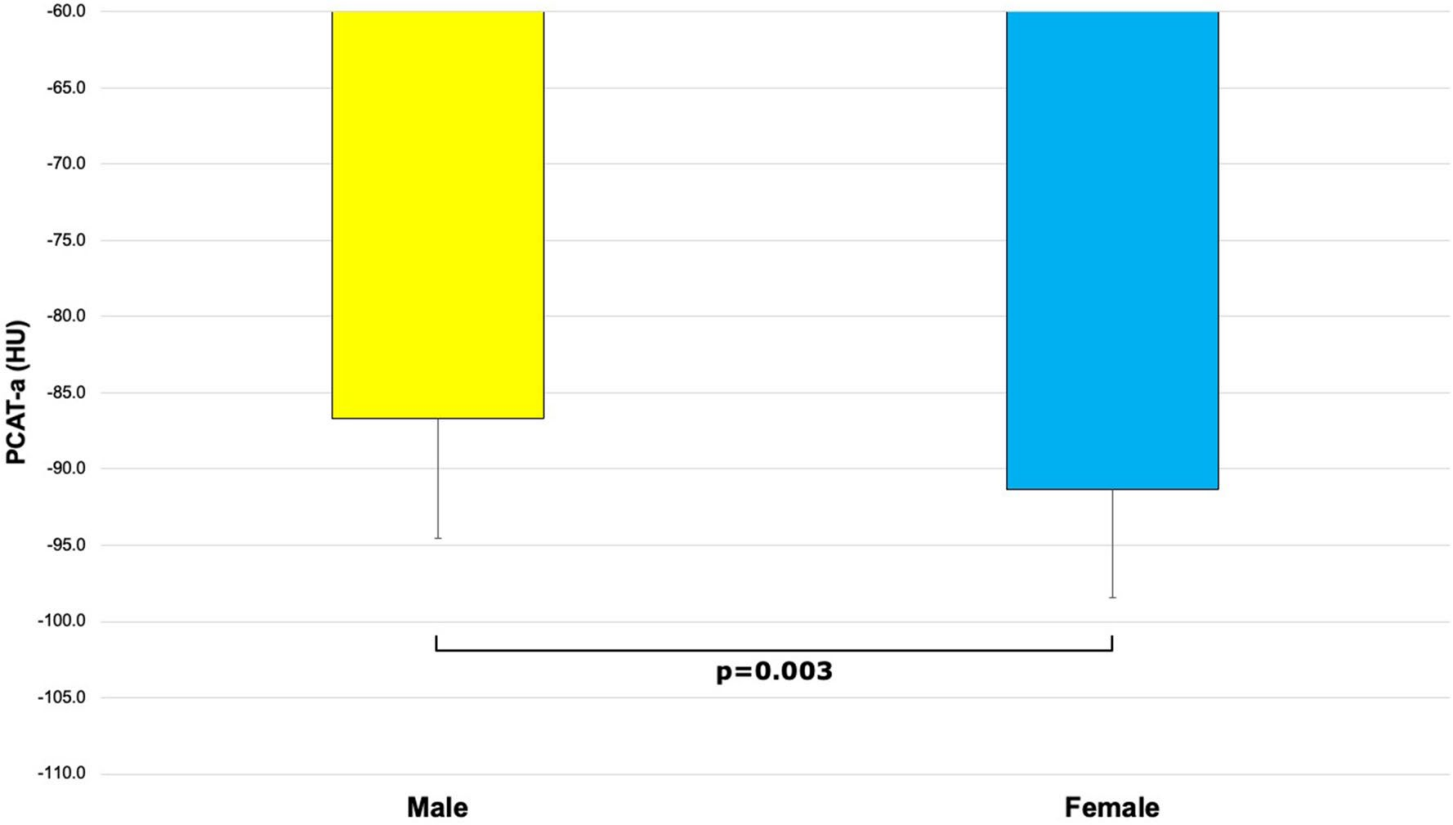
Comparison of PCAT-a in men versus women (− 86.7 ± 7.8 HU vs. − 91.3 ± 7.1 HU, p = 0.003). *PCAT-a* PCAT-attenuation*.*

Indigenous patients had numerically higher PCAT-a but this was not statistically significant (− 87.4 ± 7.6 HU vs. − 89.6 ± 8.0 HU, p = 0.133; Fig. 2). Indigenous patients had significantly higher PCAT-v (1.5 ± 0.5cm^3^ vs. 1.3 ± 0.4cm^3^, p = 0.032; Fig. S1) than controls. Male patients had significantly higher PCAT-a (− 86.7 ± 7.8 HU vs. − 91.3 ± 7.1 HU, p = 0.003; Fig. 3) and numerically higher PCAT-v (1.5 ± 0.5cm^3^ vs. 1.3 ± 0.4cm^3^, p = 0.05; Fig. S2) than female patients. There were no relationships between PCAT-a and age or cardiovascular risk factors (all p > 0.05). PCAT-v correlated with age (r = 0.256, p = 0.008) and was significantly higher in patients with hypertension (1.5 ± 0.4cm^3^ vs. 1.3 ± 0.5cm^3^, p = 0.045) and obesity (1.6 ± 0.4cm^3^ vs. 1.3 ± 0.4cm^3^, p < 0.001).

**Figure 4:**
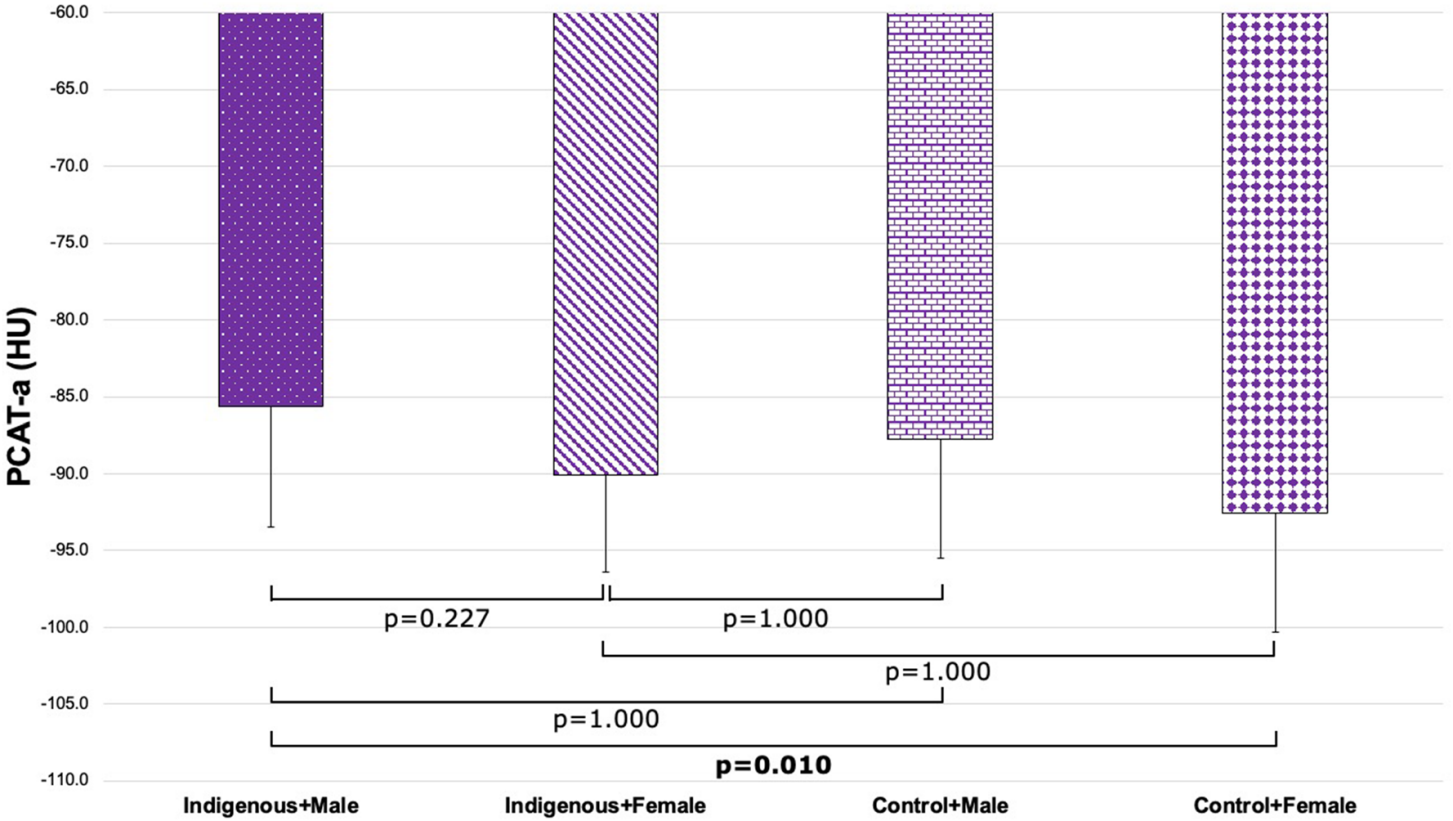
Comparison of PCAT-a across patient groups stratified by ethnicity and sex (Indigenous + Male, − 85.6 ± 7.8 HU; Indigenous + Female, − 90.1 ± 6.3 HU; Control + Male, − 87.8 ± 7.7 HU; Control + Female, − 92.5 ± 7.8 HU; p = 0.010). *PCAT-a* pericoronary adipose tissue attenuation.

**Figure 5:**
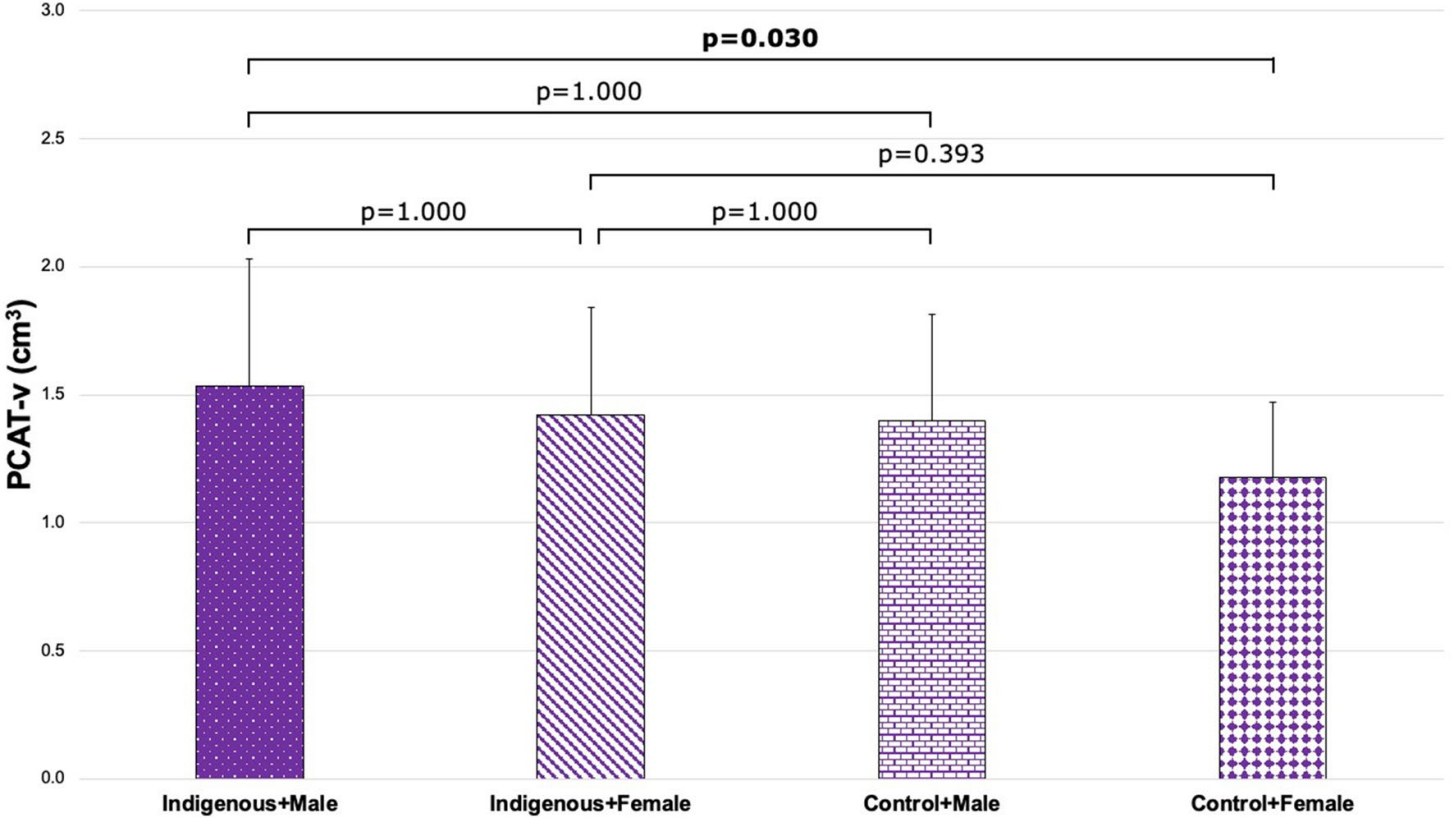
Comparison of PCAT-v across patient groups stratified by ethnicity and sex (Indigenous + Male, 1.5 ± 0.5cm^3^; Indigenous + Female, 1.4 ± 0.4cm^3^; Control + Male, 1.4 ± 0.4cm^3^; Control + Female, 1.2 ± 0.3cm^3^; p = 0.030). *PCAT-a* pericoronary adipose tissue attenuation.

To investigate the potential interaction between Indigenous status and sex, four patient groups were defined. There was a significant difference in PCAT-a across subgroups defined by Indigenous status and sex (Indigenous + Male, -85.6 ± 7.8 HU; Indigenous + Female, − 90.1 ± 6.3 HU; Control + Male, − 87.8 ± 7.7 HU; Control + Female, − 92.5 ± 7.8 HU; p = 0.010; Fig. 4). Post-hoc pairwise analysis demonstrated that Indigenous males had significantly higher PCAT-a than female controls (p = 0.008; Fig. 4). There was also a significant difference in PCAT-v across these groups (Indigenous + Male, 1.5 ± 0.5cm^3^; Indigenous + Female, 1.4 ± 0.4cm^3^; Control + Male, 1.4 ± 0.4cm^3^; Control + Female, 1.2 ± 0.3cm^3^; p = 0.030; Fig. 5). Post-hoc pairwise analysis demonstrated that Indigenous males had significantly higher PCAT-v than female controls (p = 0.018; Fig. 5).

### Subgroup of matching contrast density analysis

Subgroup analysis was performed in matched patients with matching contrast density (age: 47.6 ± 13.3; 50.0% male; n = 48). In this subgroup, Indigenous patients had significantly higher PCAT-a than controls (− 87.0 ± 8.1 HU vs. − 92.2 ± 6.9 HU, p = 0.020). There was no significant difference in PCAT-v between Indigenous patients and controls (p = 0.451). Male patients had significantly higher PCAT-a than female patients (− 87.0 ± 8.7 HU vs. − 92.2 ± 6.1 HU, p = 0.023). There was no significant difference in PCAT-v between males and females (p = 0.112).

**Figure 6:**
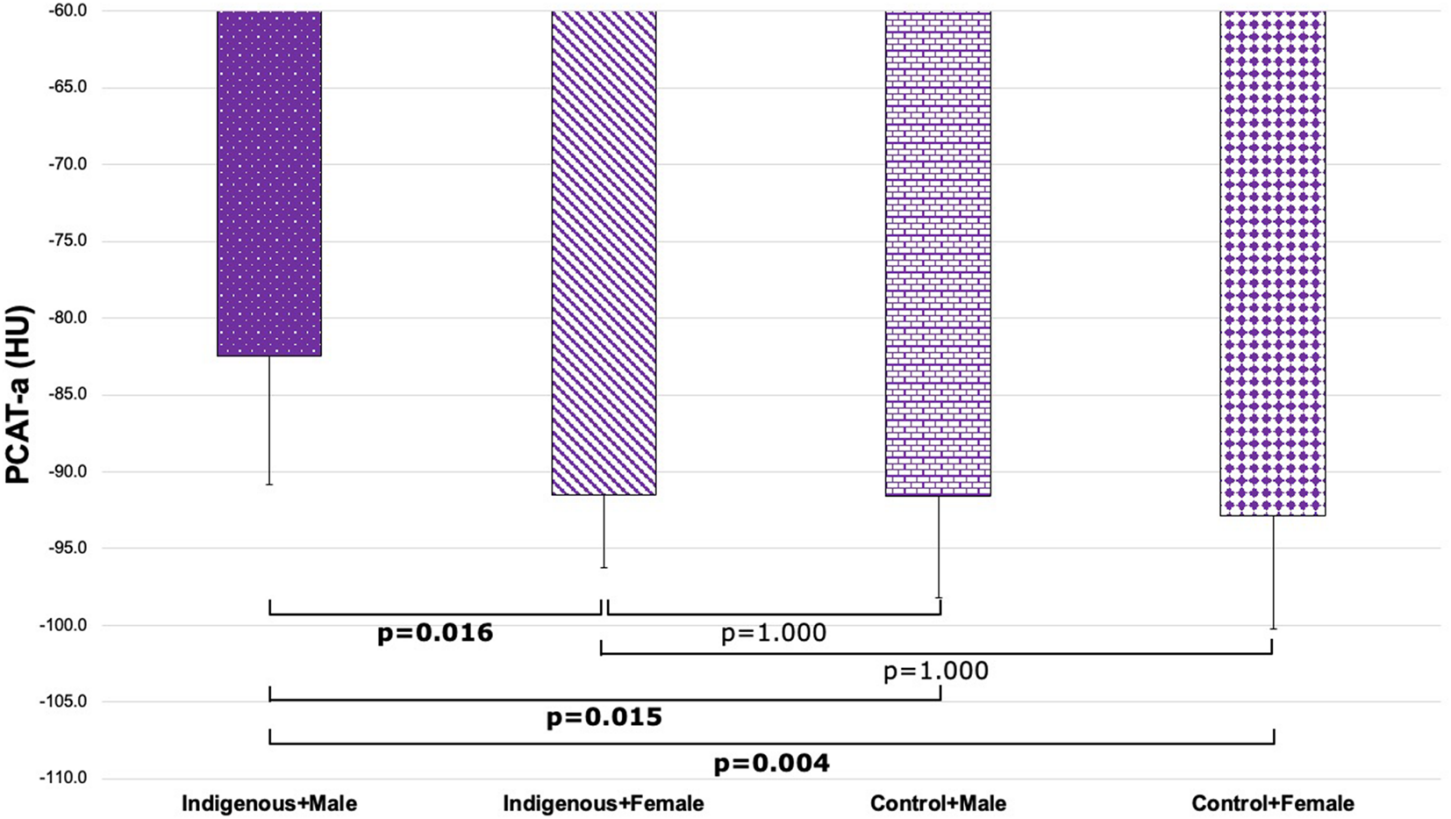
Comparison of PCAT-a across patient groups stratified by ethnicity and sex exclusively among patients with matching contrast density (Indigenous + Male, − 82.5 ± 8.3 HU; Indigenous + Female, − 91.5 ± 4.8 HU; Control + Male, − 91.6 ± 6.6 HU; Control + Female, − 92.9 ± 7.4 HU; p = 0.002). *PCAT-a* pericoronary adipose tissue attenuation.

In patient groups stratified by Indigenous status and sex, there was a significant difference in PCAT-a between groups (Indigenous + Male, − 82.5 ± 8.3 HU; Indigenous + Female, − 91.5 ± 4.8 HU; Control + Male, − 91.6 ± 6.6 HU; Control + Female, − 92.9 ± 7.4 HU; p = 0.002; Fig. 6). There was also a significant difference between Indigenous males and each group (Indigenous + Male vs. Indigenous + Female, p = 0.016; vs. Control + Male, p = 0.015; vs. Control + Female, p = 0.004; Fig. 6). There was no significant difference in PCAT-v between groups (p = 0.175).

### Multivariable regression analysis

In the overall cohort, two stepwise backward linear regression models were performed (Tables 2A and B). In the first model, PCAT-a was studied after adjustment for Indigenous status, age, sex, statin use, number of traditional risk factors and plaque burden. In this model, male sex independently associated with PCAT-a when including all covariates. In the final step of analysis, male sex and statin use associated with PCAT-a (Table 2A). In the second model, PCAT-a was studied after adjustment for Indigenous status, age, sex, statin use, number of traditional risk factors and the presence of obstructive CAD. In this model, male sex independently associated with PCAT-a when including all covariates. In the final step of analysis, age and obstructive CAD associated with PCAT-a (Table 2B).

**Table 2:** Multivariable linear regression analyses of covariates associated with PCAT-a in the overall cohort.

(A)	Model 1–first step			Final model		
	Beta	95% CI	p-value	Beta	95% CI	p-value
Indigenous Australian	2.578	− 0.488, 5.644	0.098	2.896	− 0.112, 5.904	0.059
Male sex	**3.766**	**0.466, 7.066**	**0.026**	**4.516**	**1.404, 7.628**	**0.005**
Age	− 0.117	− 0.261, 0.027	0.111	–	–	–
Statin	3.029	− 0.844, 6.901	0.124	**3.355**	**0.099, 6.612**	**0.044**
Number of c.v.R.F	− 0.255	− 1.504, 0.994	0.686	–	–	–
Plaque burden*	1.625	− 0.916, 4.166	0.207	–	–	–

(B)	Model 2–first step			Final model		
	Beta	95% CI	p-value	Beta	95%CI	p-value
Indigenous Australian	2.133	− 1.053, 5.318	0.187	–	–	–
Male sex	**3.657**	**0.374, 6.939**	**0.029**	3.052	− 0.115, 6.219	0.059
Age	− 0.117	− 0.255, 0.021	0.094	− **0.145**	− **0.273,** − **0.018**	**0.026**
Statin	2.633	− 1.341, 6.607	0.191	–	–	–
Number of c.v.R.F	− 0.145	− 1.402, 1.112	0.819	–	–	–
Obstructive CAD	3.358	− 1.049, 7.766	0.134	**5.285**	**1.427, 9.143**	**0.008**

*PCAT-a* pericoronary adipose tissue attenuation.

*Plaque Burden is defined by CT-Leaman score thresholds (CT-LeSc = 0, no plaque; CT-LeSc 0.1–8.2, low plaque burden; CT-LeSc ≥ 8.3, high plaque burden).

Significant values are in bold.

In the subgroup with matching contrast density, two similar stepwise backward linear regression models were performed (Tables 3A and B). In the first model, PCAT-a was studied after adjustment for Indigenous status, age, sex, statin use, and plaque burden. In this model, Indigenous status independently associated with PCAT-a in all steps of analysis (Table 3A). In the second model, PCAT-a was studied after adjustment for Indigenous status, age, sex, statin use and obstructive CAD. In this model, Indigenous status independently associated with PCAT-a in all steps of analysis (Table 3B).

**Table 3:** Multivariable linear regression analyses of covariates associated with PCAT-a in a subgroup with matching contrast density.

(A)	Model 1–first step			Final model		
	Beta	95% CI	p-value	Beta	95%CI	p-value
Indigenous Australian	**6.292**	**1.518, 11.066**	**0.011**	**5.930**	**1.615, 10.245**	**0.008**
Male sex	4.358	− 0.275, 8.991	0.064	3.947	− 0.373, 8.266	0.072
Age	− 0.043	− 0.247, 0.160	0.668	–	–	–
Statin	3.654	− 1.869, 9.178	0.188	–	–	–
Plaque burden*	− 0.511	− 4.564, 3.543	0.800	–	–	–

(B)	Model 2–first step			Final model		
	Beta	95% CI	p-value	Beta	95%CI	p-value
Indigenous Australian	**5.260**	**0.670, 9.851**	**0.026**	**6.110**	**1.680, 10.539**	**0.008**
Male sex	3.142	− 1.686, 7.970	0.196	–	–	–
Age	− 0.106	− 0.296, 0.084	0.266	–	–	–
Statin	2.171	− 3.110, 7.452	0.410	–	–	–
Obstructive CAD	3.351	− 3.437, 10.139	0.324	–	–	–

*PCAT-a* pericoronary adipose tissue attenuation.

*Plaque burden is defined by CT-Leaman score thresholds (CT-LeSc = 0, no plaque; CT-LeSc 0.1–8.2, low plaque burden; CT-LeSc ≥ 8.3, high plaque burden).

Significant values are in bold.

## Discussion

The findings of our study are threefold. Firstly, we found that Indigenous Australian men had significantly higher PCAT-a than non-Indigenous Australians. Secondly, men had significantly higher PCAT-a than women irrespective of Indigenous or non-Indigenous status. Lastly, PCAT-a independently associates with Indigenous Australian status after correction for age, sex, statin use, coronary plaque burden and matching contrast density.

Indigenous Australians are burdened with significant cardiovascular risk and while increases in life expectancy have been observed, reductions in cardiac mortality observed in the broader Australian population occur at a notably slower rate within the Indigenous community^1^. This highlights an increasing, rather than diminishing, divide in cardiovascular outcomes between these groups. Indeed, cardiovascular disease affects significantly younger age groups among Indigenous Australians, calling for appropriate screening to ensure timely management^10,11^. The high prevalence of traditional risk factors and chronic comorbid conditions such as chronic kidney disease, diabetes and obesity are all potent contributors to mortality, and are additive to existing social determinants impairing community engagement with preventive measures^12^. Recent evidence demonstrates that Indigenous Australians harbour a higher burden of CAD independent of risk factor incidence^13^. Strikingly, a substantial inflammatory risk may exist given reportedly high levels of CRP^7–9^, and may further account for the high rates of cardiovascular events experienced within this group. PCAT-a measured in the RCA is strongly associated with coronary events^5,14–16^ but only modestly correlates with serum biomarkers of inflammation, including CRP, among individuals with stable CAD^5,17^. This is likely owed to the specificity PCAT-a offers as a biomarker of coronary inflammation and therefore provides a useful means of evaluating its role in elevated CAD risk amongst Indigenous Australians.

The hypothesis that excess visceral adiposity may contribute to cardiovascular risk specifically within this group has been explored by Sun et al.^3^, who found increased EAT-volume within a group of Indigenous Australians compared to a matched group of Caucasians. We expand upon these findings by investigating coronary inflammation as shown by attenuation differences within PCAT, a subset of EAT. Among patients exposed to identical CCTA protocols and with matching contrast density at the right coronary ostium, we found that Indigenous Australians had significantly higher PCAT-a than age- and sex-matched non-Indigenous controls. To our knowledge, ours is the first study to report increased coronary inflammation in Indigenous Australians, and more broadly within a given ethnic group. Goeller et al.^18^ performed a comparative analysis between matched South Asian, East Asian and Caucasian cohorts, but found no difference in PCAT-a based on ethnicity alone. To some degree our results echo these findings, in that analysis in our overall cohort of matched individuals, Indigenous Australian ethnicity alone produced only a numerical difference in PCAT-a compared to non-Indigenous controls. In contrast, our subgroup analysis demonstrated significantly higher PCAT-a in Indigenous Australians compared to controls. This subgroup was characterised not only by a high matching contrast density but also by equal proportions of male and female patients in both Indigenous Australian patients and controls, which may have minimised the strong impact of sex on analysis.

We matched patients by sex to account for potential biological differences in CAD pathophysiology between men and women. Interestingly, stratifying patients by both sex and Indigenous or non-Indigenous status revealed that Indigenous Australian men harbour significantly higher coronary inflammation than all other groups. It has been previously reported that Indigenous Australian status significantly mitigates sex differentials in cardiovascular outcomes, particularly among younger individuals, as shown by a lower sex-specific ratio of age-standardised acute and 2-year mortality rate following a first myocardial infarction (MI) in comparison to large non-Indigenous samples^11,19^. Notably, amongst younger Indigenous Australians (age 25–54 years), male sex still associated with over a twofold increased rate of post-MI mortality^11,19^. Our finding of increased PCAT-a in Indigenous men falls within this age range and therefore concords with the literature, suggesting Indigenous Australian status in conjunction with male sex produces an even higher inflammatory risk within the coronary vasculature.

Indeed, male sex was in itself a prominent risk factor associated with increased PCAT-a in all our analyses. Secondary evidence from numerous studies highlights the potential impact played by male sex on PCAT attenuation^15,18,20–22^. Where no ethnic differences in PCAT-a were found, the aforementioned study by Goeller et al. showed a significantly higher attenuation in men^18^. Sugiyama et al. found that PCAT-a was significantly associated with male sex as well as obstructive plaque in the RCA^20^. A recent study by Bengs et al. reported the prognostic value of PCAT-a for MACE was hampered by an interaction with sex^15^. Moreover, we have previously reported male sex resulted in increased PCAT-a even amongst patients with vulnerable coronary plaque^21^. A striking similarity between these disparate studies is the numerical difference in attenuation between males and females – approximately 5–6 HU in every study that evaluated PCAT-a within the proximal RCA^15,18,20,21^. Our study confirms these findings that among age-matched individuals male sex still produces significantly higher PCAT-a of a similar magnitude, adding to an already mounting body of evidence implicating coronary inflammation as a potential contributor to the heightened coronary risk faced by men. Indigenous Australian men already harbour greater burden of CAD^13,23^, and our findings highlight that even after adjustment for plaque burden, heightened coronary inflammation in this group may further precipitate poorer outcomes.

We also matched patients by age, to account for the notion that CAD affects a younger distribution of Indigenous versus non-Indigenous Australians. It is plausible that if we confined our study cohort to a younger age range, we may have found a more profound difference in PCAT-a among Indigenous Australians that is congruent with an earlier onset of coronary events. Indeed, though statistically insignificant, PCAT-a correlated negatively with age. Inclusion of a wide range in age may have subjected our results to shifting levels of coronary inflammation, reflecting perhaps individual variance in plaque vulnerability and stability rather than differences in inflammation owing primarily to ethnicity.

Protocol-related factors are an important consideration in all CCTA analysis, and numerous technical parameters may manifest in potentially confounding imaging artefact if not properly controlled. Though a multi-centre study, our entire study cohort was evaluated using the same CCTA scanners and protocol. We further controlled for any effects pertaining to partial volume averaging in our subgroup with a matching high level of contrast density. Hell et al. demonstrated significantly lower PCAT-a in the distal compared to proximal LAD, consistent with a decline in luminal contrast density^24^. Ma et al. showed significantly different PCAT-a owing to tube voltage ranging from 70 to 120 kV, but no significant difference amongst patients scanned at 100 and 120 kV^22^. Our analysis showed that in patients with strong luminal contrast filling and scanned at only 100 or 120 kV, strong relationships between increased PCAT-a and Indigenous status as well as male sex are found in comparison to our overall cohort, further highlighting the potentially confounding impact of contrast attenuation on PCAT analysis.

PCAT-v was also higher in Indigenous Australian men when compared to either Indigenous Australian women, or non-Indigenous controls of either sex. However, in our subgroups with matching contrast density no difference in PCAT-v was found between these groups. PCAT-v has been associated with coronary plaque^25,26^ and with coronary vasospasm^27,28^ but, to our knowledge, it has not been explored in relation to ethnicity. PCAT-v was significantly increased among males in our study, and in Indigenous Australian males compared to female controls. The accumulation of PCAT mass would be consistent with the previous observation of increased EAT-v in this group^3^. While the significance of increased PCAT-v in CAD is yet to be determined, it is clear that in combination with PCAT-a these findings highlight adverse cardiac adiposity as a potential player in the cardiovascular risk endured by men, particularly those of Indigenous descent.

Our study has a number of limitations. We were unable to adjust for a positive family history of IHD, due to the lack of family data in a significant proportion of patients within our Indigenous cohort. Similarly, additional parameters, such as systolic blood pressure, required to calculate the Framingham risk score as an alternative measure of cardiovascular risk were incomplete, rendering the evaluation of such metrics beyond the scope of our analysis. However, otherwise complete risk factor profiles were obtained for both our Indigenous and non-Indigenous cohorts. We also note that ongoing statin therapy was prevalent in both cohorts, but in relatively equal proportions (31.3% vs. 33.3%, p = 0.822; Table 1), and was also corrected for in all multivariable analyses. Our control cohort was comprised of a range of ethnicities, including patients of South Asian, East Asian and Caucasian descent, but little difference in PCAT-a has been reported between these groups^18^. There was also a numerically higher proportion of Indigenous patients who had categorically high plaque burden as well as obstructive CAD, and due to the retrospective nature of our sampling the impact of plaque burden and morphology on PCAT-a must not be understated. Nevertheless, both obstructive CAD and categorical plaque burden were among the covariates that were adjusted for in all multivariable analysis, but future studies may provide further evidence of coronary inflammation among patient cohorts with only no plaque or stable CAD. Finally, as our study cohort was comprised of only symptomatic individuals referred for CCTA, our findings should not be generalised to the broader Indigenous Australian population.

Indigenous Australian men have increased coronary inflammation evidenced by increased PCAT attenuation on CCTA. Male sex is strongly associated with increased PCAT attenuation. Indigenous Australian status is associated with increased PCAT attenuation after adjustment for age, male sex, statin use and plaque burden among a subgroup with matching contrast density. Our findings suggest coronary inflammation may be an important contributor to the adverse cardiac profile exhibited by Indigenous Australians.

## Methods

### Patients

All data was retrospectively obtained from patients undergoing CCTA for suspected CAD in 2018 at two hospitals. A study population of 108 patients, comprised of 54 Indigenous Australian and 54 non-Indigenous patients (controls), was derived from the Royal Darwin Hospital (Tiwi, NT, Australia) and Monash Medical Centre (Clayton, VIC, Australia). Patients were matched by age and sex. Clinical data including statin use, cardiovascular risk factors and BMI were obtained via questionnaire at the time of CCTA. Statin use was defined as any statin therapy at the time of CCTA. Hypertension was defined as systolic blood pressure > 140 mmHg and diastolic blood pressure > 90 mmHg at CCTA, or diagnosis/treatment for hypertension. Hypercholesterolaemia was defined as fasting total cholesterol > 6.2 mmol/L, high-density lipoprotein cholesterol < 1.0 mmol/L, low-density lipoprotein cholesterol > 3.4 mmol/L, or treatment for hypercholesterolaemia. Type II diabetes mellitus was defined as HbA1c ≥ 6.5% or treatment with glucose-lowering medications. Body mass index (BMI) was calculated as the weight divided by height in square metres (kg/m^2^), and obesity was defined as BMI ≥ 30 kg/m^2^. The number of risk factors was defined as the sum of all traditional risk factors per patient excluding family history of IHD, which was omitted from analysis due to incomplete data within the Indigenous Australian cohort.

### CCTA protocol

All patients were scanned on 320-detector row scanner (Aquilion Vision; Canon Medical Systems Corporation, Otawara, Japan) at Royal Darwin Hospital and Monash Medical Centre using the same protocol as previously described^29^. All scans were performed using prospective ECG-gating during a single-breath hold. A standard heart rate of 60 bpm was maintained through beta-blockade. Contrast-enhancement was mediated by intravenous administration of Omnipaque 350 (60-90 mL) at a rate of 5 mL/s. All scans were subject to scan parameters of tube voltage 100-120 kV, tube current 300-500 mA, collimation 320 × 0.5 mm, gantry rotation time 275 ms and temporal resolution 175 ms. Acquisition parameters adhered to a limited range for all patients in our cohort: 300-500 mA tube current, 100-120 kV tube voltage, collimation 320 × 0.5 mm, gantry rotation time 275 ms and temporal resolution 175 ms. Images were collated and reconstructed using a reconstruction kernel (FC03). All scans were performed in conjunction with department-specific protocol and other relevant guidelines^30,31^.

### Coronary plaque classification

The presence and extent of coronary plaque was adjudicated by two cardiologists with at least two years of CCTA experience as part of routine clinical evaluation. The segment involvement score (SIS) defines the number of coronary segments with plaque irrespective of the degree of stenosis^32^. Segment stenosis score (SSS) was calculated as the sum of stenosis scores for each plaque-affected segment as measured on a four-point scale: 0, no stenosis; 1, 1–24% stenosis; 2, 25–49% stenosis; 3, 50–74% stenosis; 4, ≥ 75% stenosis^33^. Obstructive CAD was defined as ≥ 50% stenosis in at least one vessel segment. CT-Leaman score (CT-LeSc) provides the sum of a numerical score quantifying plaque burden for each coronary segment, with correction for obstructive or non-obstructive stenosis, morphology, vessel localisation and coronary artery dominance. Three categories of plaque burden were defined based on previously established CT-LeSc thresholds: 0, no plaque; 0.01–8.29, low plaque burden; and ≥ 8.3, high plaque burden^34^. Additionally, the Coronary Artery Disease – Reporting and Data System (CADRADS) score was used to stratify patients based on severity of stenosis of the most obstructive coronary lesion.

### Classification of patient groups by ethnicity and sex

The interaction of Indigenous status and sex was investigated by categorising patients into four groups: Indigenous + male patients; Indigenous + female patients; control + male patients; and control + female patients.

### Pericoronary adipose tissue segmentation

PCAT was defined as all voxels between − 190 to − 30 Hounsfield Units (HU) on routine CCTA within a predefined volume of interest around the proximal right coronary artery (RCA). We studied PCAT attenuation (PCAT-a) and PCAT volume (PCAT-v) within all patients, which were measured in HU and cm^3^, respectively. All PCAT segmentation was performed on a validated semi-automated software package (AutoPlaque v2.5) by trained operators with at least one-year experience with said software (JY, EL), using a previously described methodology. Briefly, the operator manually identifies and marks the RCA. A region of interest (ROI) marker is placed at the ostium to define the attenuation of a normal blood pool within the contrast enhanced coronary vasculature. A proximal 40 mm segment, beginning 10 mm from the vessel ostium, is selected. The vessel adventitia is traced to demarcate surrounding adipose tissue from the vessel wall. The software then automatically detects mean attenuation and total volume of PCAT extending up to 3 mm from the vessel wall, which approximates the typical diameter of the RCA.

### Classification of patient subgroup with matching contrast density

It has been established that contrast density affects PCAT-a due to partial volume averaging^24^. To control for this potential confounder, we identified a subgroup of patients consisting entirely of patients with matching contrast density, defined as luminal attenuation of ≥ 500 HU at the coronary ostium. This subgroup was comprised only of age- and sex-matched pairs.

### Statistical analysis

Mean and standard deviation were used to describe parametric data, while median and interquartile range (IQR) described non-parametric data. PCAT-a and PCAT-v were compared between Indigenous Australians and control groups, and between male and female groups, using the independent samples *t* test. Comparison of PCAT-a and PCAT-v across Indigenous and sex-derived groups was performed using the one-way ANOVA, and the Bonferroni post-hoc test was used to derive significance of pairwise comparisons between select groups. The Chi-square test was performed to study categorical risk factors (male sex, hypertension, hypercholesterolaemia, type II diabetes, smoking status and obesity) and statin use between Indigenous patients and controls. Pearson and Spearman correlation analyses were used to study PCAT against continuous covariates, namely age and BMI. All covariates achieving p < 0.2 on univariable analysis were corrected for in subsequent adjusted analysis. Multivariable linear regression analysis was performed to study the effect of Indigenous Australians on PCAT-a after correction for selected covariates. For all analysis, a two-sided p-value of p < 0.05 was considered statistically significant. All statistical analysis was performed using SPSS (version 27).

### Ethics approval

This study was performed in lines with the principles of the Declaration of Helsinki. Full ethics approval was granted by the Monash Health Human Research Ethics Committee (12 March 2020/No. EC00382) and the Menzies School of Health Research Ethics Committee (9 December 2019/EC00153).

### Consent to participate

Informed consent as obtained from all individual participants included in the study.

### Supplementary Information

Supplementary Figure S1.

Supplementary Figure S2.

Supplementary Legends.

## Data Availability

All data was obtained for clinical purposes. In the interest of maintaining confidentiality for all participants in the study, the datasets analysed will not be made publicly available. All inquiries regarding access to study data may be directed to DW.
