# Supplementary figures and images for: Pericoronary adipose tissue attenuation on coronary computed tomography angiography associates with male sex and Indigenous Australian status
Source: Sci Rep. 2023 Sep 19;13:15509. doi: 10.1038/s41598-023-41341-9 (PMC10509231; doi:10.1038/s41598-023-41341-9)

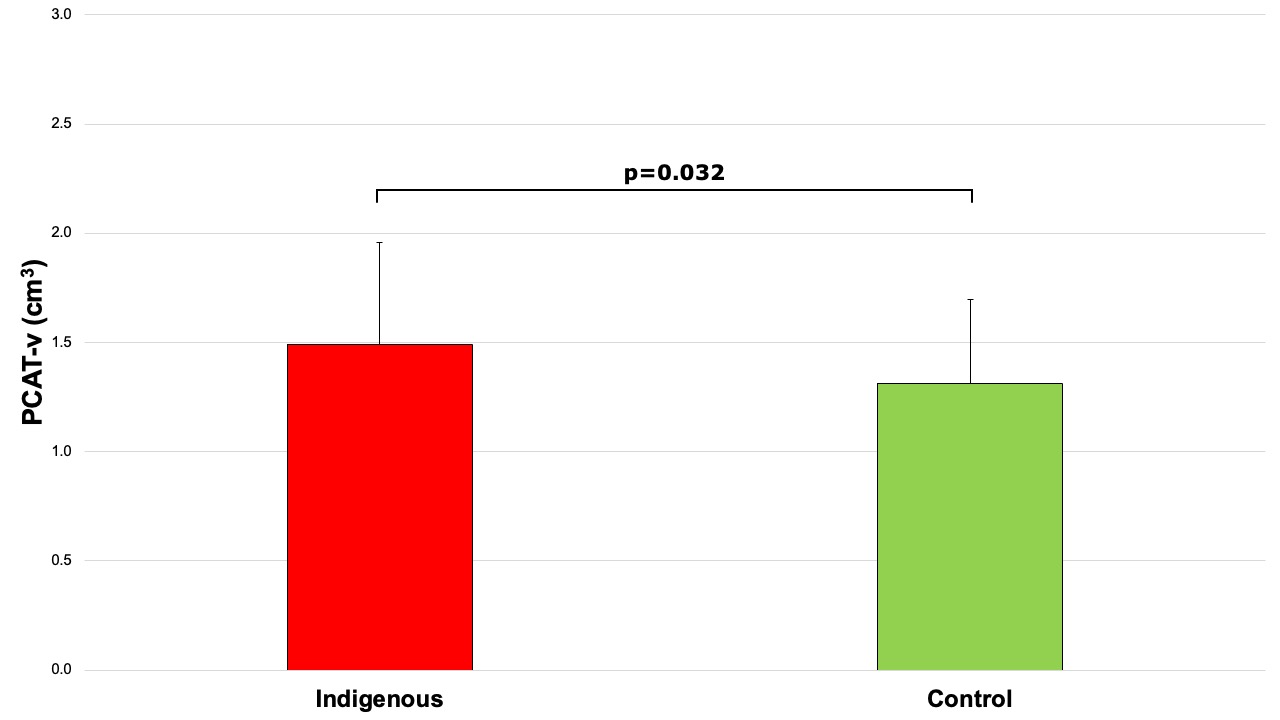

Supplement: Supplementary file 1 — Supplementary Figure S1. [file 41598_2023_41341_MOESM1_ESM.jpg]

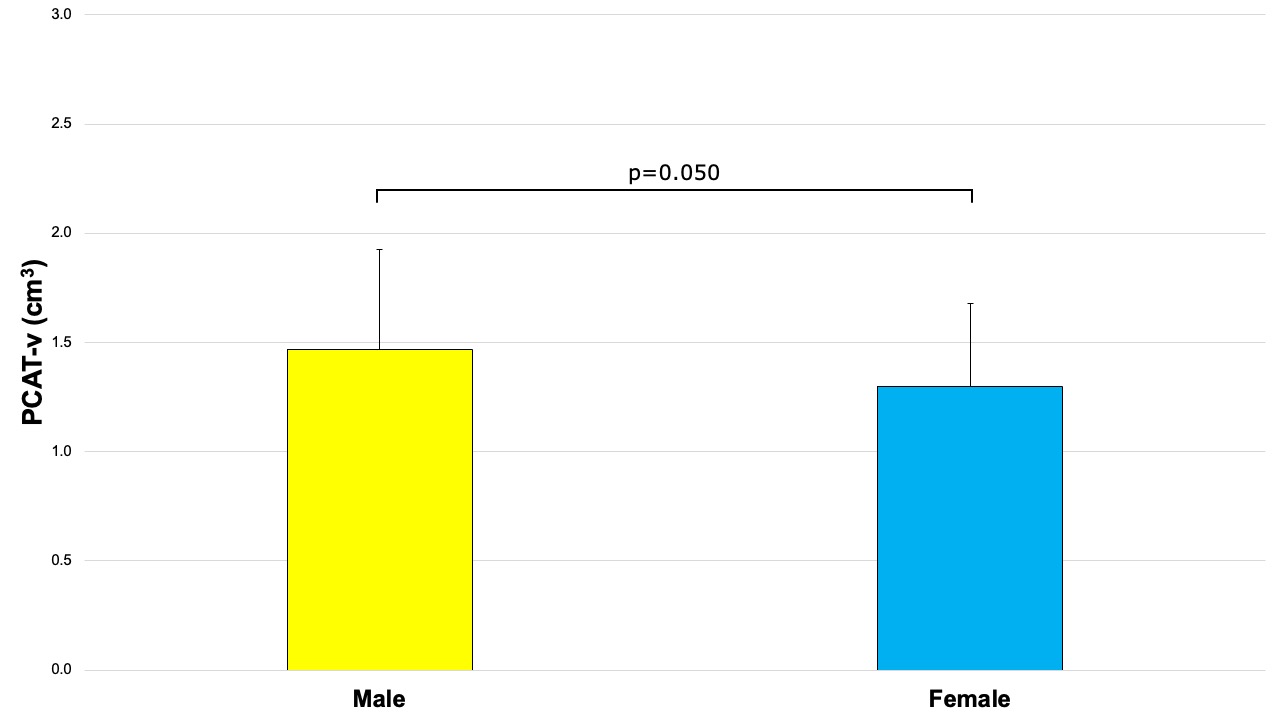

Supplement: Supplementary file 2 — Supplementary Figure S2. [file 41598_2023_41341_MOESM2_ESM.jpg]
