# Supplementary material for: Pericoronary adipose tissue attenuation on coronary computed tomography angiography associates with male sex and Indigenous Australian status
Source: Sci Rep. 2023 Sep 19;13:15509. doi: 10.1038/s41598-023-41341-9 (PMC10509231; doi:10.1038/s41598-023-41341-9)
Supplement: Supplementary file 3 — Supplementary Legends. [file 41598_2023_41341_MOESM3_ESM.docx]

***SUPPLEMENTARY FIGURES***

**Figure S1**. Comparison of PCAT-v in Indigenous Australians versus controls (1.5±0.5cm^3^ vs. 1.3±0.4cm^3^, p=0.032). *PCAT-v, pericoronary adipose tissue volume*.

**Figure S2**. Comparison of PCAT-v in males versus females (1.5±0.5cm^3^ vs. 1.3±0.4cm^3^, p=0.05). *PCAT-v pericoronary adipose tissue volume.*
